# Vitamin D deficiency in patients with retinal vein occlusion: a systematic review and meta-analysis

**DOI:** 10.1186/s40942-024-00571-3

**Published:** 2024-07-27

**Authors:** Kimia Daneshvar, Mohammadreza Akhlaghi, Shila Iranpour, Matin Irajpour, Mohsen Pourazizi

**Affiliations:** 1 Isfahan Eye Research Center, Isfahan University of Medical Sciences, Isfahan, Iran; 2 Isfahan Eye Research Center, Department of Ophthalmology, Isfahan University of Medical Sciences, Isfahan, Iran; 3 Department of Ophthalmology, Feiz Hospital, Modares St, Isfahan, Iran

**Keywords:** Vitamin D, 25-Hydroxyvitamin D, Retinal Vein Occlusion, Meta-analysis

## Abstract

**Background:**

This review aims to substantiate the correlation between vitamin D and retinal vein occlusion (RVO) within the medical literature.

**Method:**

A systematic review and meta-analysis were conducted in PubMed, SCOPUS, Web of Science, and Embase until December 10th, 2023. A meticulous literature search was undertaken to identify and analyze all observational-analytical papers reporting vitamin D levels in RVO patients. The principal outcome measures centered on the comparative assessment of vitamin D levels between patients with RVO (cases) and those devoid of RVO (controls). The protocol was registered in PROSPERO (code: CRD42024499853).

**Results:**

A total of six relevant studies consisting of 589 participants were included in this meta-analysis. The results indicated a significant association between vitamin D deficiency and increased risk of RVO (Odds ratio = 14.51; 95% CI: [1.71, 122.59], *P* = 0.014); and patients with RVO exhibited a significant decrease in serum vitamin D levels by 1.91ng/mL (95% CI: [-2.29, -1.54], *P* < 0.001). Moreover, there was no significant difference observed in vitamin D levels between central RVO (CRVO) and branch RVO (BRVO) subtypes (*P* = 0.63).

**Conclusion:**

RVO patients have more vitamin D deficiency than healthy controls. These results contribute to the growing body of evidence highlighting the intricate role of vitamin D supplementation as both a prophylactic and a treatment strategy in RVO.

**PROSPERO registration identifier:**

: CRD42024499853.

**Supplementary Information:**

The online version contains supplementary material available at 10.1186/s40942-024-00571-3.

## Introduction

Retinal vein occlusion (RVO) is a major cause of visual loss, in fact after diabetic retinopathy it is the most common retinal vascular disease, which predominantly affects older adults [1]. Although studies have detected higher prevalence of RVO among certain ethnicities, but it was mainly attributed to the differences in the prevalence and distribution of RVO risk factors [2, 3]. Recent studies surmised that atherosclerosis and traditional cardiovascular risk factors including hypertension [4], older age [5], hypertriglyceridemia, renal dysfunction [6], diabetes mellitus [7], and higher body mass index [8] are the shared risk factors associated with retinal vein occlusion. Moreover, the presence of carotid plaques and increase in brachial-ankle pulse wave velocity as subclinical determinants of atherosclerosis were considered contributors to RVO [9].

Vitamin D, has a potentially remarkable role in maintaining vascular endothelium. Several studies have demonstrated the association of vitamin D deficiency with cerebrovascular incidents [10] and coronary artery disease [11]. Vitamin D deficiency has been linked to higher rates of hypertension, especially considering inhibition of renin-angiotensin-aldosterone system by vitamin D [12]. Other studies have suggested direct effect of hypovitaminosis D on increased vascular resistance with respect to the role of vitamin D in promoting nitric oxide synthesis [13]. Furthermore vitamin D inhibits the production of inflammatory cytokines including tumor necrosis factor alpha (TNF-a) and interleukin 6 (IL-6), and thereby modulates the inflammatory responses within the arterial wall [14]. In the interim, vitamin D has the capacity to attenuate the oxidative stress and reduce the production of reactive oxygen species [15]. Vitamin D is also considered a potential regulator of lipid metabolism. The activation of certain genes responsible for cholesterol efflux is facilitated by vitamin D, which aids in the removal of cholesterol from macrophages and prevents the formation of foam cells; leading to maintenance and stabilization of plaques and decreased likelihood of cardiovascular events [16]. Additionally, it can impact the function of enzymes involved in lipid metabolism, which may help regulate lipid profiles and maintain the balance between LDL and HDL [17].

There is growing evidence to suggest that maintaining adequate levels of vitamin D may be beneficial for preventing and managing RVO [18]. Therefore, the following systematic review, aims to explore and analyze the existing research on the relationship between Vitamin D levels and the incidence of RVO.

## Materials and methods

Following systematic review and meta-analysis adhered to the PRISMA guidelines and was prospectively registered with the International Prospective Register of Systematic Reviews (PROSPERO) with the following identifier: CRD42024499853. This study was also approved by Institutional Review Board of Isfahan University of Medical Sciences (IR.ARI.MUI.REC.1403.063). The study protocol included predefined search strategies, criteria for assessing study quality, and a statistical plan. Furthermore, outcome measures, inclusion, and exclusion criteria were defined a priori to ensure methodological transparency and analytical rigor.

### Search strategy

An electronic search was conducted across PubMed, Scopus, Web of science, and Embase databases up to December 10th, 2023. Employed keywords were “retinal vein occlusion” OR “RVO” OR “retinal vein obstruction” OR “retinal vein thrombosis” and “vitamin D” OR “25-hydroxyvitamin D” OR “25-hydroxycholecalciferol” OR “calcifediol” OR “calcidiol” OR “25OHD” OR “25OH vitamin D” (Table S1). No limitations were imposed on data or language. Titles, abstracts, and full texts of retrieved documents were systematically assessed. Additionally, a meticulous examination of reference lists from relevant papers was performed to include articles presenting data on vitamin D levels in the context of RVO for inclusion in our analytical literature.

### Inclusion and exclusion criteria

The process of study selection was undertaken independently by two reviewers (KD and SI). Discrepancies were resolved through discussion, and where consensus was challenging, opinion of a third expert reviewer (MP) was sought for resolution. This systematic review and meta-analysis included studies conducted on human subjects aged 18 years or older, focusing on the correlation of vitamin D levels and RVO. The inclusion criteria strictly selected participants devoid of underlying diseases or other concurrent retinal disorders, while excluding those taking medications that could affect vitamin D levels. Notably, there was no restrictions based on race, geographical region, or language. Also, studies were included irrespective of matching or non-matching methodologies. Publications such as opinion pieces, case reports, review articles, book chapters, letters, and studies lacking substantive data pertaining to the association between vitamin D and RVO were excluded.

### Data extraction

Two reviewers (KD and SI) independently performed the extraction of data from eligible articles. The process utilized a Microsoft Excel spreadsheet (Microsoft, Redmond, Washington, USA), intricately designed following the Cochrane meta-analysis guidelines and tailored to suit the specific needs of this review. The extracted data included study characteristics (first author’s name, journal, year of publication, country of study performance, study design, and sample size), participants’ characteristics (age and gender), and the outcome measures (mean serum vitamin D levels in RVO patients of either type (CRVO and BRVO) and control group, as well as the prevalence of vitamin D deficiency in both groups). We converted vitamin D levels to ng/mL in studies where they were originally reported in nmol/mL. To ensure accuracy and prevent omissions, a subsequent validation of the extracted data was undertaken by the expert reviewer (MP).

### Quality assessment of studies

The quality of the included studies was evaluated using the Joanna Briggs Institute (JBI) checklist for case-control and randomized controlled trial study designs [19]. This checklist consists of 10 questions with four possible response options: yes, no, uncertain, and not applicable. The assessment was conducted independently by two reviewers. For the scoring, a point was assigned for each “yes” response, while “no”, “uncertain”, and “not applicable” responses received 0 points. A cutoff of 7 points was defined as the minimum score for inclusion in the analyses. (Table S2).

### Effect size

In order to perform our meta-analysis continuous variables are represented using standard mean difference (SMD) and 95% CI, and binary variables are conveyed through log odds ratio (Log OR) and 95% CI.

### Statistical analysis

We conducted a meta-analysis using the Stata 17 (StataCorp LP, College Station, TX, USA) and comprehensive meta-analysis 3.7 software (Biostat, Englewood, USA) to summarize the findings of the studies. The DerSimonian and Laird random-effects models were utilized for the summary of SMD and Log OR. To assess the heterogeneity, statistical tests such as the Cochrane’s Q and I² tests were employed. In order to control the heterogeneity level, subgroup analyses was performed based on matching methodology regarding sex, seasonality, diet, hospital or healthy controls, vitamin D measurement assay and country of study. The level of statistical significance was defined as *p* < 0.05 for the Q-test. Sensitivity analysis was conducted to assess the impact of single study on the overall summary estimation. Publication bias was evaluated both statistically, using Egger’s and Begg’s tests, and visually inspecting of the funnel plot.

## Results

### Study characteristics

**Fig. 1:**
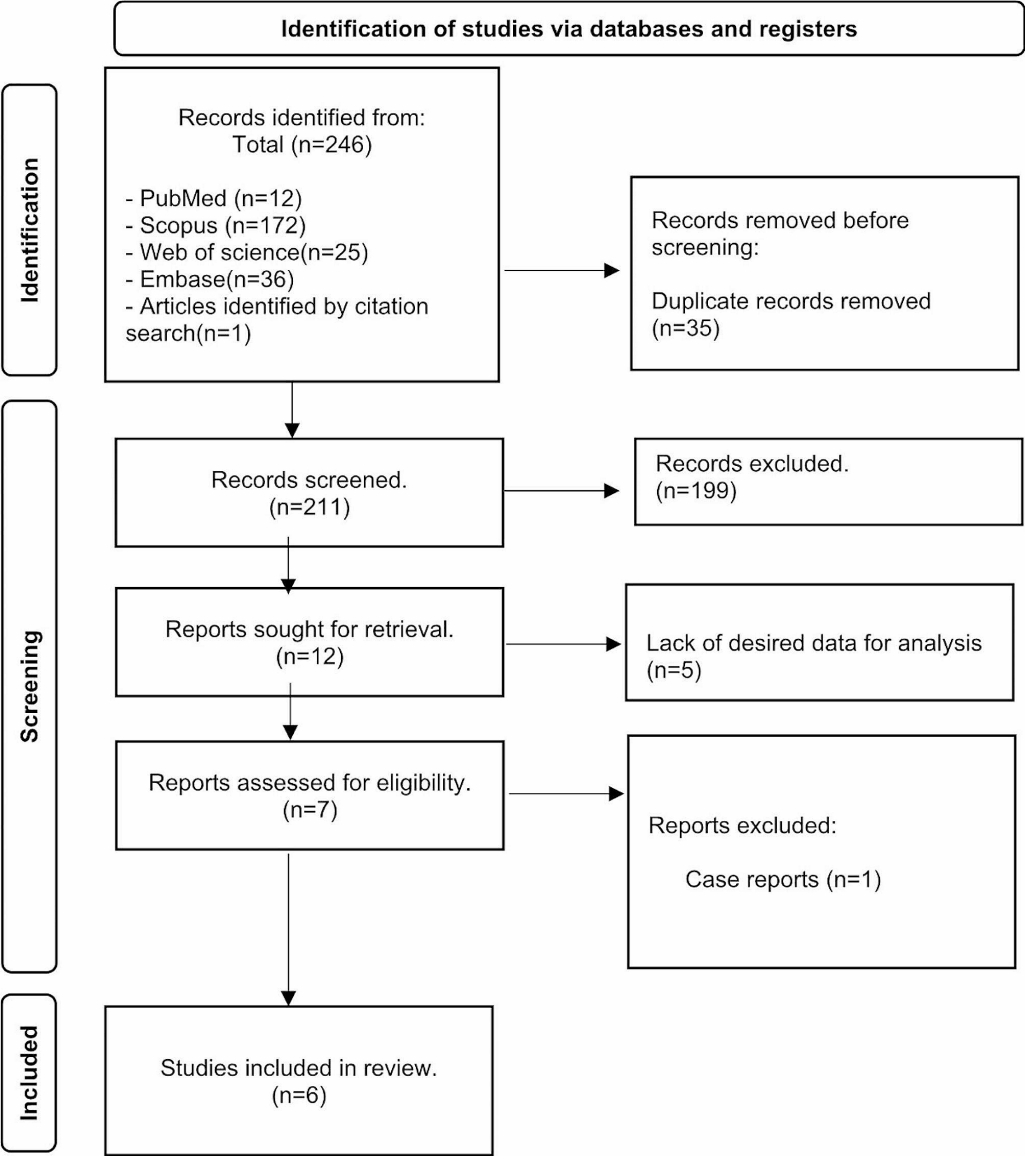
PRISMA diagram describing the study selection process for the systematic review and meta-analysis

Initially 246 potentially relevant papers were identified. Of which 35 were duplicates and were removed. During title and abstract screening, 199 studies did not fulfill the inclusion criteria, therefore they were not included in the study. The full texts of remaining 12 papers underwent a meticulous review. Subsequently, one paper [20], identified as a case report, was excluded, while five other papers were omitted due to lack of desired data for analysis [21–25]. Finally, 5 case-control studies [26–30] and one prospective interventional study [18] were included in our meta-analysis. The flow diagram illustrating the study selection is presented in Fig. 1.

Table 1 presents the primary information extracted from the six included studies. The studies, were published between 2016 and 2023, and were conducted in Europe [26] and Asia [18, 27–30]. Study sample sizes ranged from 35 to 68 cases of RVO, encompassing a total of 289 cases of which 154 and 135 were diagnosed with CRVO and BRVO respectively. Control groups consisted of 300 individuals. Controls were comprised of both healthy individuals and hospital-based subjects without RVO. Five studies reported mean levels of serum vitamin D in both RVO and matched control groups [26–30]. Mean age of the patients was 63.75 ± 3.01 years. All studies had enrolled patients with RVO duration of less than three months. Additionally, all of them measured serum levels of 25 (OH) vitamin D following a 12-hour fasting period. The range of differences between mean levels of vitamin D in RVO patients and controls were 1.88 ng/mL and 16.40 ng/mL, respectively. With the exception of the study conducted by Epstein et al. [26], each study incorporated in the analysis reported statistically significant lower levels of vitamin D among RVO patients compared to their respective control group. Vitamin D levels within the subtypes of RVO (CRVO and BRVO) had been elucidated in five studies [18, 27–30]. In each of included studies, when comparing vitamin D levels between BRVO and CRVO patients, no statistically significant difference was observed in the intergroup comparison. While all studies considered levels below 20ng/mL as deficient, Karimi et al [18], used a higher threshold, defining deficiency as levels below 30ng/mL. Four studies had investigated the prevalence of vitamin D deficiency within the context of RVO compared to the control group [26–28, 30]. The prevalence of vitamin D deficiency among RVO patients were within the range of 20–95%. All the studies included in the analysis received high quality scores according to the JBI quality assessment.

**Table 1:** Summary of the studies

First Author, year	Country	Age		Vitamin D Method	Sample size (Male)		RVO type		Serum Vitamin D		Serum Vitamin D		Vitamin D deficiency%	
		RVO	Control		RVO	Control	BRVO	CRVO	RVO	Control	BRVO	CRVO	RVO	Control
Epstein ^25^, 2016	Sweden	74.1 ± 8.7	73.7 ± 7.6	ND	68(36)	140(84)	0	68	22.0 ± 1.4	23.9 ± 0.36	ND	22.1 ± 1.4	51.40%	39.30%
Oli ^26^, 2017	India	60.2 ± 9.7	60.7 ± 9.9	MS/MS	40(30)	40(26)	26	14	13.7 ± 4.6	23.0 ± 2.9	12.8 ± 3.9	15.4 ± 5.3	95%	12.50%
Karimi ^30^, 2022	Iran	62 ± 8	ND	ND	68(38)	ND	42	26	28.6 ± 14.5	ND	28.5 ± 14.7	28.8 ± 14.6	ND	ND
Muttar ^29^, 2023	Iraq	62.9% >50 years	74.3%>50 years	ELFA	35(20)	35(17)	14	21	14.2 ± 5.2	22.7 ± 4.4	15.2 ± 5.5	13.2 ± 5.0	82.85%	22.85%
Bhanot ^28^, 2023	India	62 ± 10.4	60 ± 9.7	ELFA	43(32)	50(33)	30	13	19.1 ± 9.1	32.3 ± 10.1	20.6 ± 9.7	15.7 ± 6.8	ND	ND
Kandambeth ^27^, 2023	India	60.1 ± 10.8	60.2 ± 10.8	MS/MS	35(ND)	35(ND)	23	12	21.4 ± 4.9	37.8 ± 11.8	21.7 ± 4.6	20.9 ± 5.6	20%	0%

RVO, retinal vein occlusion; BRVO, branch retinal vein occlusion; CVRO, central retinal vein occlusion; MS/MS, Tandem mass spectrometry ; ELFA, enzyme-linked fluorescence assay; ND, not defined

### Meta-analysis results

**Fig. 2:**
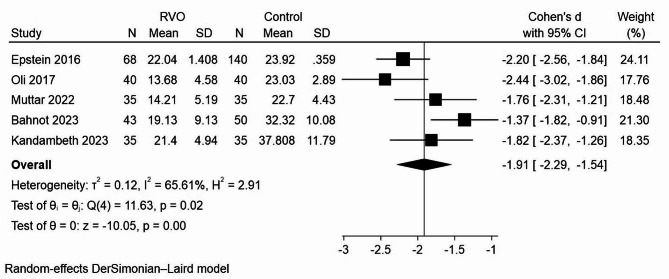
Forest plot assessing standard mean difference in serum 25(OH) vitamin D levels between RVO patients and the control group

The mean serum vitamin D levels in patients with RVO and the control group were recorded as 19.71 ± 1.917 and 26.89 ± 1.18, respectively. Meta-analysis results from these studies revealed a statistically significant overall SMD of − 1.91 ng/mL in serum vitamin D levels between RVO patients and controls (95% CI: -2.29, -1.54, *P* < 0.001) (Fig. 2). However, significant heterogeneity was observed (I^2^ = 65.61%, *P* = 0.02), as depicted in Galbraith plot (Figure S1). In order to assess the influence of different exclusion criteria on the overall risk estimate, we conducted sensitivity and subgroup analyses to discern potential sources of heterogeneity within this meta-analysis. In the assessment of the mean difference of vitamin D between RVO group and the control, the study carried by Bhanot et al. [29], emerged as the prominent contributor to heterogeneity in this meta-analysis. Upon exclusion of this specific study, the observed heterogeneity reduced to 26.39%, yielding a SMD of -2.07 (95% CI -2.36, -1.78) (Figure S2).

After implementing either age, sex, diet, seasonality matching between RVO and control group, a subsequent subgroup analysis was conducted (Table S3). In a subgroup analysis, which was based on the selection of control group from the community, in studies with population-based controls significantly lower mean levels of vitamin D were observed in RVO patients compared to the studies which had with hospital-based control groups (SMD= -2.27; 95% CI: [-2.57, -1.96]; *P* < 0.001; SMD= -1.61; 95% CI: [-1.90, -1.31]; *P* < 0.001; respectively) (Table S3, Figure S3). Studies with seasonal variation matching methodology recorded significantly lower vitamin D levels in RVO patients compared to studies without this matching methodology (SMD= -2.27; 95%CI -2.57, -1.96; *P* < 0.001) (Table S3, Figure S4). There was no statistically significant subgroup effect regarding vitamin D assessment method, study country, matching methodology regarding sex and diet (Table S3,4, and 5). Moreover, meta-regression did not find any correlation between SMD of vitamin D and sample size, patients’ age, male percentage, and country latitude (Table S6).

Despite a subtle asymmetry observed in the funnel plot, both Egger’s and Begg’s tests detected no statistically significant indication of publication bias (*P* = 0.94 and 0.46; respectively) (Figure S9).

**Fig. 3:**
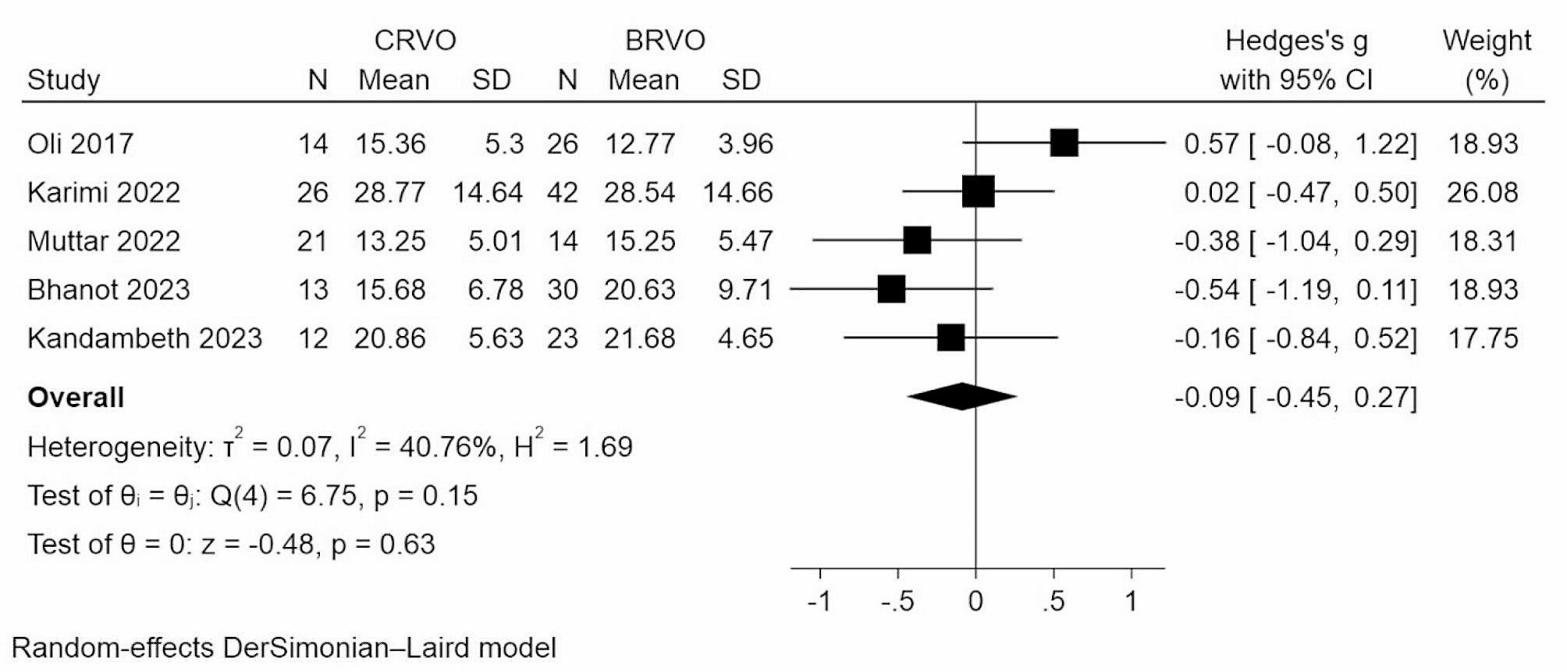
Forest plot assessing standard mean difference in serum 25(OH) vitamin D levels between RVO patients and the control group in CRVO and BRVO patients

Furthermore, a meta-analysis was conducted to assess the potential variations in vitamin D levels across different subtypes of retinal vein occlusion. Five studies reported mean levels of vitamin D according to the type of RVO [18, 27–30]. While the analysis indicated slightly higher mean vitamin D levels in BRVO patients as compared with CRVO individuals, the results did not reach statistical significance threshold (SMD= -0.09; *P* = 0.63; I^2^ = 40.76%) (Fig. 3).

### Results of the meta-analysis of the prevalence of vitamin D deficiency in RVO

**Fig. 4:**
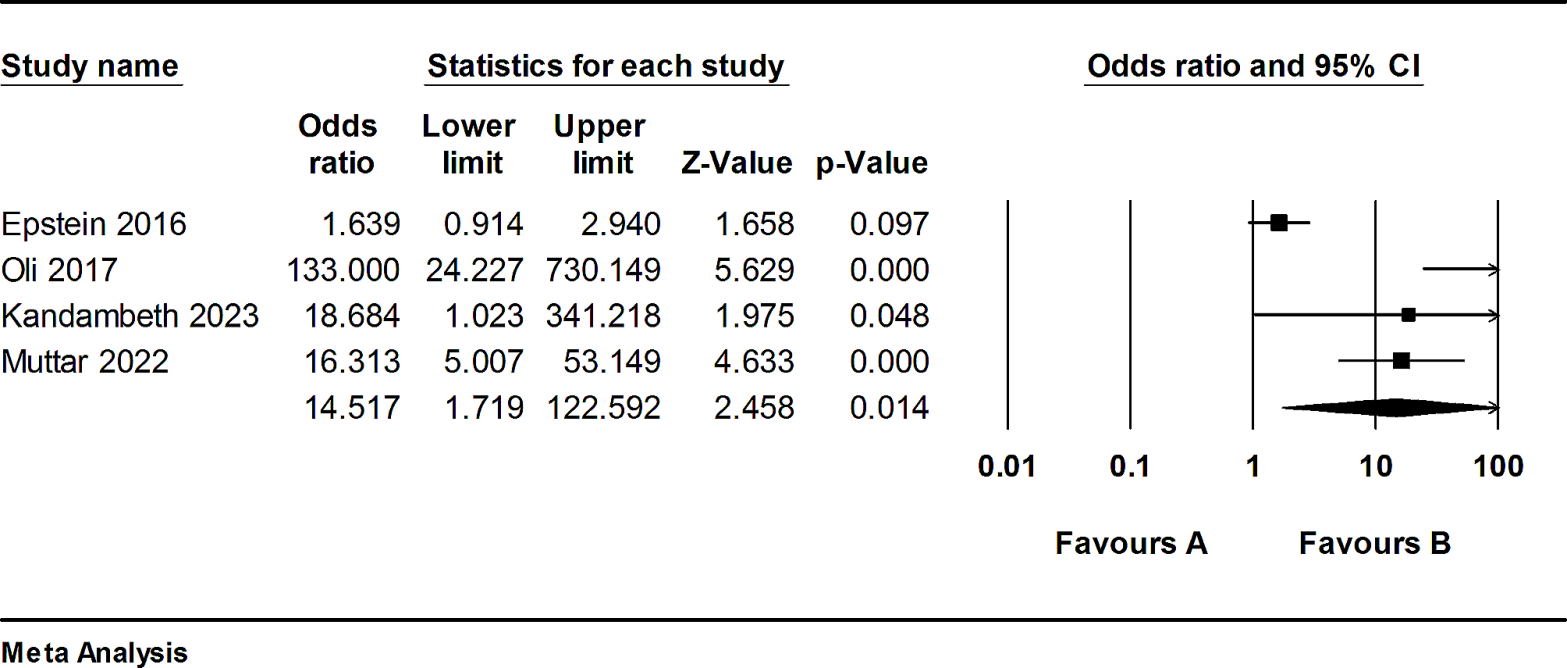
A forest depicting the odds ratio of vitamin D deficiency in RVO patients

In four studies [26–28, 30], the prevalence of vitamin D deficiency in patients was reported alongside matched control groups. We conducted a meta-analysis, integrating the reported rates of vitamin D deficiency from these studies and estimating the corresponding odds ratio. The results demonstrated a statistically significant higher vitamin D deficiency among patients as opposed to the controls (Odds ratio = 14.51; 95% CI: [1.71, 122.59], *P* = 0.014) (Fig. 4).

A sensitivity analysis was undertaken to explore potential sources of heterogeneity, revealing higher results with decreased heterogeneity upon exclusion of the study conducted by Epstein et al. [26] (Odds ratio = 34.93; 95% CI: [8.03, 151.94]; *P* < 0.001; I^2^ = 50.71%) (Figure S10).

## Discussion

This meta-analysis marks the first investigation into the correlation between serum vitamin D status and retinal vein occlusion. The consolidated outcomes reveal a substantially lower serum levels of 25(OH)D levels among RVO patients compared to the controls. Given the lack of significant difference in serum vitamin D levels between CRVO and BRVO subtypes in the included studies, the meta-analysis also did not reveal a significant distinction between the two groups. Furthermore, the results of meta-analysis, incorporating findings from four studies reporting vitamin D deficiency prevalence, suggest a significant association between the prevalence of vitamin D deficiency and RVO. Surprisingly, no individuals with RVO exhibited sufficient levels of vitamin D in any of the included studies reporting the prevalence of vitamin D deficiency [27, 28, 30].

Vitamin D deficiency has been identified in numerous cardiovascular and cerebrovascular incidents, hinting at a potential association with disorders affecting retinal vasculature [31]. The correlation between vitamin D deficiency, hypertension, and increased vascular resistance prompts consideration of potential confounding factors related to RVO [32, 33]. Nevertheless, the study Kandambeth et al [28], revealed no significant disparity in vitamin D levels between hypertensive and non-hypertensive RVO patients, indicating a direct influence of vitamin D on RVO that appears independent of being normotensive or hypertensive. However, one noteworthy limitation in the majority of previous primary studies was the lack of reported data on the number of hypertensive and normotensive subjects. Future works should mention the prevalence of hypertension in their study population. As for dyslipidemia, the association of hyperlipidemia and RVO has been documented in numerous studies [6, 9, 34]. The role of vitamin D in development of dyslipidemia is still a controversial topic. Although many observational studies have found evidence of a strong association, interventional studies have not yet substantiated these claims [35].

In the sensitivity analysis performed during meta-analysis on the odds ratio of vitamin D deficiency, the study conducted by Epstein et al [26] was found as a potential source of heterogeneity. With the exception of the study conducted by Epstein et al [26], all included studies yielded consistent findings, indicating a significant difference in vitamin D levels between patients and controls. In essence, the study by Epstein et al., didn’t have enough statistical power to conclusively confirm a relationship between vitamin D levels and the risk of central retinal vein occlusion due to less pronounced observed difference. Nevertheless, the study successfully demonstrated a significant difference in vitamin D levels in the study group of patients aged < 75 years compared to the controls. Intriguingly, older patients had more favorable vitamin D levels [26].

Despite the fact that none of the studies assessed vitamin D levels at the onset of retinal vein occlusion, a three-month interval from the onset of the disease appears to have no significant impact on the relationship between vitamin D status and RVO.

The study by Karimi et al. [18], highlights a noteworthy finding, demonstrating a significant reduction in central macular thickness (CMT) in CRVO patients treated with intravitreal bevacizumab (IVB) along with oral vitamin D supplementation compared to those who received IVB alone. This combined treatment approach exhibited substantial improvement in best-corrected visual acuity (BCVA). These results underscore the potential benefits of integrating vitamin D supplementation with intravitreal therapy in managing RVO [18]. These findings collectively emphasize the intricate interplay of vitamin D deficiency and subsequent inflammatory process in influencing the severity and progression of RVO.

There is a complex interplay of risk factors associated with retinal vein occlusion, including diabetes mellitus, hypertension, dyslipidemia, cardiovascular disease, and smoking [36], and vitamin D deficiency is implicated in the development of mentioned risk factors [37, 38]. Previous literature have surmised that CRVO and BRVO could be associated with different systemic diseases; for instance, arterial hypertension was significantly more prevalent in BRVO patients [36]. In the present study, no statistically remarkable difference was noted in comparing BRVO and CRVO subgroups; therefore, the role of vitamin D and its association with both conditions demand further investigations. Indeed, it’s unclear whether low vitamin D directly contributes to retinal vein occlusive disease, or if other confounding factors are involved in this relationship as well. Our meta-analysis revealed a significantly lower vitamin D levels in patients diagnosed with RVO compared to controls. The controlled analysis of included studies revealed an insignificant difference concerning comorbid conditions between RVO patients and the control group, effectively managing confounding effects on vitamin D [26–28, 30].

## Conclusions

Our meta-analysis established a noteworthy association between vitamin D deficiency and retinal vein occlusion. However, to comprehensively understand the potential roles and mechanisms, further investigations especially clinical trials are essential. Specifically, exploring the impact of vitamin D supplementation on CMT and the prognosis of BCVA in RVO patients will contribute invaluable insights to improve treatment strategies.

### Electronic supplementary material

Below is the link to the electronic supplementary material.

Supplementary Material 1

Supplementary Material 2

## Data Availability

The data supporting the findings of this study are accessible upon request from the corresponding authors.

## Abbreviations

RVO: Retinal Vein Occlusion
CRVO: Central Retinal Vein Occlusion
BRVO: Branch Retinal Vein Occlusion
TNF-a: Tumor Necrosis Factor Alpha
IL-6: Interleukin 6
PRISMA: Preferred Reporting Items for Systematic Reviews and Meta-Analyses
PROSPERO: International Prospective Register of Systematic Reviews
SMD: Standard Mean Difference
CI: Confidence Interval
JBI: Joanna Briggs Institute
CRD: Centre for Reviews and Dissemination
OR: Odds Ratio
IVB: Intravitreal Bevacizumab
CMT: Central Macular Thickness
BCVA: Best-Corrected Visual Acuity
LDL: Low-Density Lipoprotein
HDL: High-Density Lipoprotein
25OHD: 25-Hydroxyvitamin D
