# Supplementary material for: Vitamin D deficiency in patients with retinal vein occlusion: a systematic review and meta-analysis
Source: Int J Retina Vitreous. 2024 Jul 27;10:52. doi: 10.1186/s40942-024-00571-3 (PMC11282712; doi:10.1186/s40942-024-00571-3)
Supplement: Supplementary file 1 — Supplementary Material 1 [file 40942_2024_571_MOESM1_ESM.docx]

**Table S1.** Search strategies for each database.

|  | |
| --- | --- |
| **Database** | **Search string** |
| PubMed | (("retinal vein thrombosis") OR ("retinal vein occlusion") OR ("retinal vein obstruction") OR (RVO) OR ("branch retinal vein occlusion") OR ("central retinal vein occlusion")) AND (("Vitamin D" OR "25-hydroxyvitamin D" OR "25-hydroxycholecalciferol" OR "calcifediol" OR "calcidiol" OR "25OHD" OR "25OH vitamin D" OR "vitamin D deficiency")) AND (1990/01/01:2023/12/10[dp]) |
| Embase | ('retinal vein thrombosis'/exp OR 'retinal vein thrombosis' OR 'retinal vein occlusion'/exp OR  'retinal vein occlusion' OR 'retinal vein obstruction'/exp OR 'retinal vein obstruction' OR rvo OR  'branch retinal vein occlusion'/exp OR 'branch retinal vein occlusion' OR 'central retinal vein  occlusion'/exp OR 'central retinal vein occlusion') AND ('vitamin d'/exp OR 'vitamin d' OR '25-  hydroxyvitamin d'/exp OR '25-hydroxyvitamin d' OR '25-hydroxycholecalciferol'/exp OR '25-  hydroxycholecalciferol' OR 'calcifediol'/exp OR 'calcifediol' OR 'calcidiol'/exp OR 'calcidiol' OR  '25ohd' OR '25oh vitamin d' OR 'vitamin d deficiency'/exp OR 'vitamin d deficiency') AND  [1990-2024]/py |
| Scopus | ALL ( ( "retinal vein thrombosis" ) OR ( "retinal vein occlusion" ) OR ( "retinal vein obstruction" ) OR ( rvo ) OR ( "branch retinal vein occlusion" ) OR ( "central retinal vein occlusion" ) ) AND ALL ( "Vitamin D" OR "25-hydroxyvitamin D" OR "25-hydroxycholecalciferol" OR "calcifediol" OR "calcidiol" OR "25OHD" OR "25OH vitamin D" OR "vitamin D deficiency" ) |
| Web of science | (ALL=(("retinal vein occlusion") OR ("retinal vein obstruction") OR (" retinal vein thrombosis")  OR (RVO) OR ( "branch retinal vein occlusion") OR (" central retinal vein occlusion") )) AND  ALL=(("Vitamin D" OR "25-hydroxyvitamin D" OR "25-hydroxycholecalciferol" OR  "calcifediol" OR "calcidiol" OR "25OHD" OR "25OH vitamin D" OR "vitamin D deficiency")) |

**Table S2.** Quality assessment of the studies

| 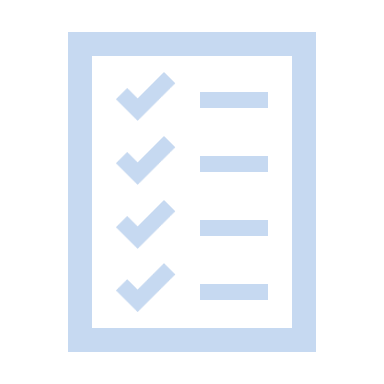Articles | Q1 | Q2 | Q3 | Q4 | Q5 | Q6 | Q7 | Q8 | Q9 | Q10 | Q11 | Q12 | Q13 |
| --- | --- | --- | --- | --- | --- | --- | --- | --- | --- | --- | --- | --- | --- |
| Case control |  |  |  |  |  |  |  |  |  |  |  |  |  |
| Bhanot 2023 | 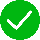 | 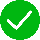 | 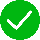 | 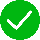 | 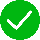 | 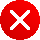 | 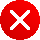 | 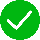 | 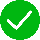 | 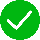 | - | - | - |
| Epstein 2016 | 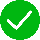 | 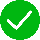 | 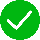 | 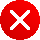 | 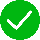 | 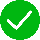 | 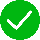 | 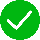 | 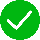 | 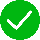 | - | - | - |
| Oli 2017 | 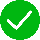 | 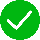 | 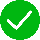 | 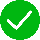 | 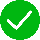 | 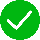 | 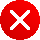 | 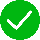 | 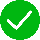 | 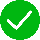 | - | - | - |
| Kandambeth 2023 | 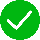 | 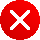 | 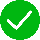 | 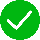 | 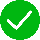 | 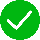 | 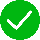 | 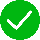 | 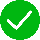 | 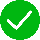 | - | - | - |
| Muttar 2023 | 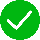 | 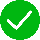 | 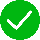 | 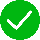 | 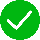 | 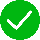 | 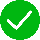 | 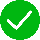 | 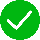 | 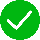 | - | - | - |
| Randomized clinical trial |  |  |  |  |  |  |  |  |  |  |  |  |  |
| Karimi 2022 | 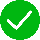 | 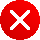 | 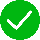 | 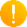 | 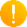 | 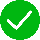 | 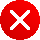 | 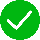 | 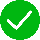 | 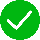 | 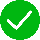 | 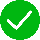 | 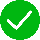 |

JBI Critical Appraisal Tool. Each question is answered in three ways: (Yes =
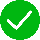
 , No =
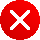
, and Unclear =
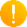
), '-' indicates not applicable or not assessed.

**Table S3**. Sub-Group Analysis based on the matching methodology

| **Variables/Group vs Control** | **All Eligible Studies** | | | **Matched** | | | **No available matching methodology** | | | |  | |
| --- | --- | --- | --- | --- | --- | --- | --- | --- | --- | --- | --- | --- |
|  | **Standardized Mean Difference (95% CI)** | | ***p* value** | **Standardized Mean Difference (95% CI)** | | ***p* value** | **Standardized Mean Difference (95% CI)** | | ***p* value** | | ***p* value between** | |
| **Seasonality** |  | |  |  | |  |  | |  | |  | |
| RVO vs Controls | -1.91 [-2.29 to -1.54] | | **<0.001** | ﻿-2.27 [-2.57 to -1.96] | | **<0.001** | -1.61 [-1.90 to -1.31] | | **<0.001** | | **<0.001** | |
| **Control population** |  | |  | Population-based | |  | Hospital-based | |  | |  | |
| RVO vs Controls | -1.91 [-2.29 to -1.54] | | **<0.001** | ﻿-2.27 [-2.57 to -1.96] | | **<0.001** | -1.61 [-1.90 to -1.31] | | **<0.001** | | **<0.001** | |
| **Sex** |  | |  |  | |  |  | |  | |  | |
| RVO vs Controls | -1.91 [-2.29 to -1.54] | | **<0.001** | -1.94 [-2.40 to -1.47] | | **<0.001** | -1.82 [-2.37 to -1.26] | | **<0.001** | | 0.75 | |
| **Diet** |  | |  |  | |  |  | |  | |  | |
| RVO vs Controls | -1.91 [-2.29 to -1.54] | | **<0.001** | -2.09 [-2.76 to -1.43] | | **<0.001** | -1.81 [-2.33 to -1.29] | | **<0.001** | | 0.50 | |
|  | |  | |  |  | | |  | |  | |  |

RVO, Retinal vein occlusion

**Table S4.** Sub-Group Analysis based on the Country

| **Variables/Group vs Control** | **All Eligible Studies** | | | **Asian countries** | | | **European countries** | | | |  | |
| --- | --- | --- | --- | --- | --- | --- | --- | --- | --- | --- | --- | --- |
|  | **Standardized Mean Difference (95% CI)** | | ***p* value** | **Standardized Mean Difference (95% CI)** | | ***p* value** | **Standardized Mean Difference (95% CI)** | | ***p* value** | | ***p* value**  **between** | |
|  |  | |  |  | |  |  | |  | |  | |
| RVO vs Controls | -1.91 [-2.29 to -1.54] | | **<0.001** | ﻿-1.82 [-2.26 to -1.38] | | **<0.001** | -2.20 [-2.56 to -1.84] | | **<0.001** | | 0.20 | |
|  | |  | |  |  | | |  | |  | |  |

RVO, Retinal vein occlusion

**Table S5.** Sub-Group Analysis based on the vitamin D assessment method

| **Variables/Group vs Control** | **All Eligible Studies** | | | **MS/MS** | | | **ELFA** | | | |  | |
| --- | --- | --- | --- | --- | --- | --- | --- | --- | --- | --- | --- | --- |
|  | **Standardized Mean Difference (95% CI)** | | ***p* value** | **Standardized Mean Difference (95% CI)** | | ***p* value** | **Standardized Mean Difference (95% CI)** | | ***p* value** | | ***p* value**  **between** | |
|  |  | |  |  | |  |  | |  | |  | |
| RVO vs Controls | -1.91 [-2.29 to -1.54] | | **<0.001** | ﻿-2.12 [-2.74 to -1.51] | | **<0.001** | -1.51 [-1.91 to -1.15] | | **<0.001** | | 0.11 | |
|  | |  | |  |  | | |  | |  | |  |

RVO, Retinal vein occlusion; MS/MS, Tandem mass spectrometry ; ELFA, enzyme-linked fluorescence assay;

**Table S6.** Results of univariate meta-regression on SMD of vitamin D

| **Variables** | **Sample Size** | | **Sex**  (Male Percentage) | | **Age** | | | **Country latitude** | | |
| --- | --- | --- | --- | --- | --- | --- | --- | --- | --- | --- |
|  | **β coefficient** | **p value** | **β coefficient** | **p value** | | **β coefficient** | **p value** | | **β coefficient** | **p value** |
| **SMD of vitamin D**  (RVO vs Controls) | -.0105618 | 0.504 | -.0161118 | 0.735 | | -.0273475 | 0.518 | | -.0087923 | 0.474 |

RVO; Retinal vein occlusion; SMD, Standard mean difference
