# Supplementary material for: Vitamin D deficiency in patients with retinal vein occlusion: a systematic review and meta-analysis
Source: Int J Retina Vitreous. 2024 Jul 27;10:52. doi: 10.1186/s40942-024-00571-3 (PMC11282712; doi:10.1186/s40942-024-00571-3)
Supplement: Supplementary file 2 — Supplementary Material 2 [file 40942_2024_571_MOESM2_ESM.docx]

**Supplemental figure 1**. Galbraith plot of standard mean difference of serum vitamin D levels between RVO patients and controls.


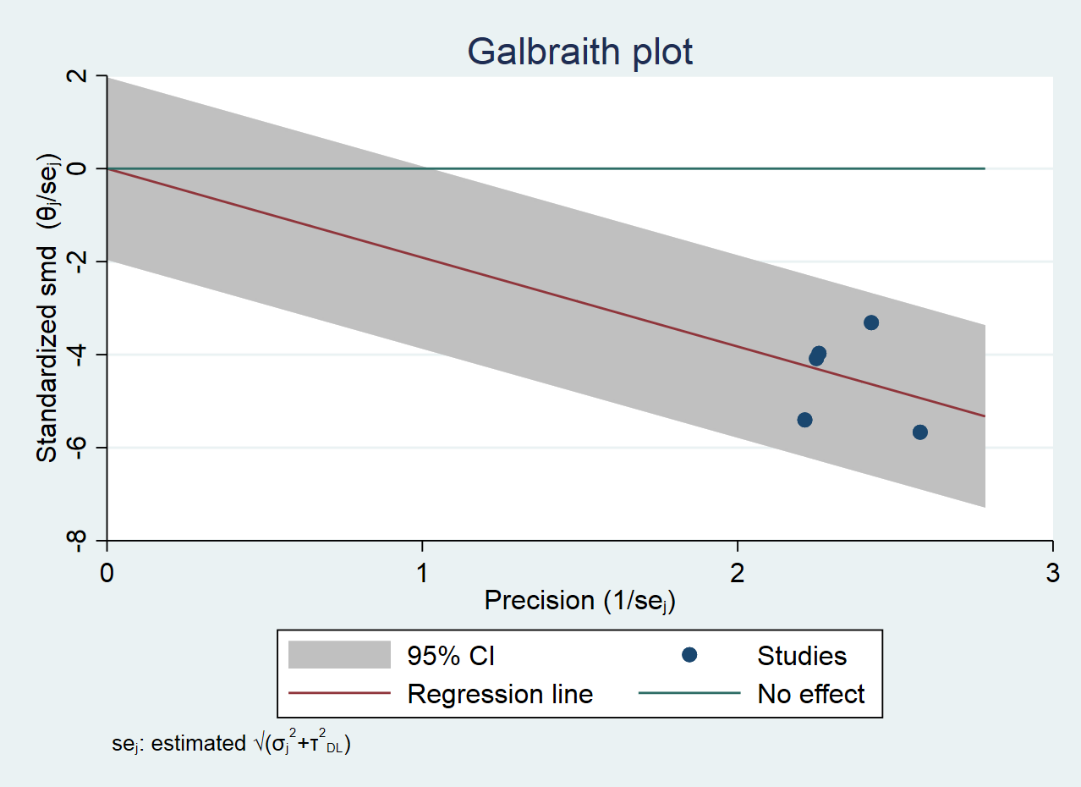


**Supplemental figure 2**. Forest plot of sensitivity analysis.


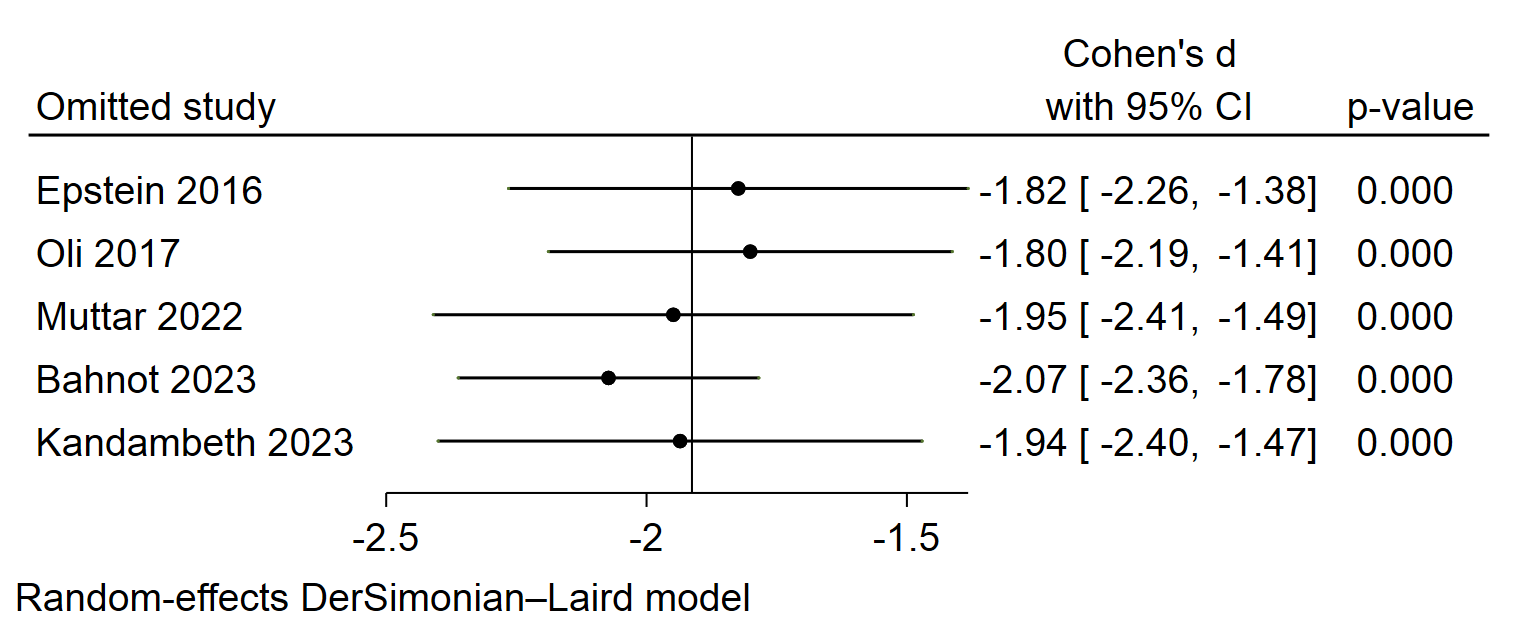


**Supplemental figure 3**. Forest plot of standard mean difference of vitamin D between RVO and controls according to the matching methodology based on control group population


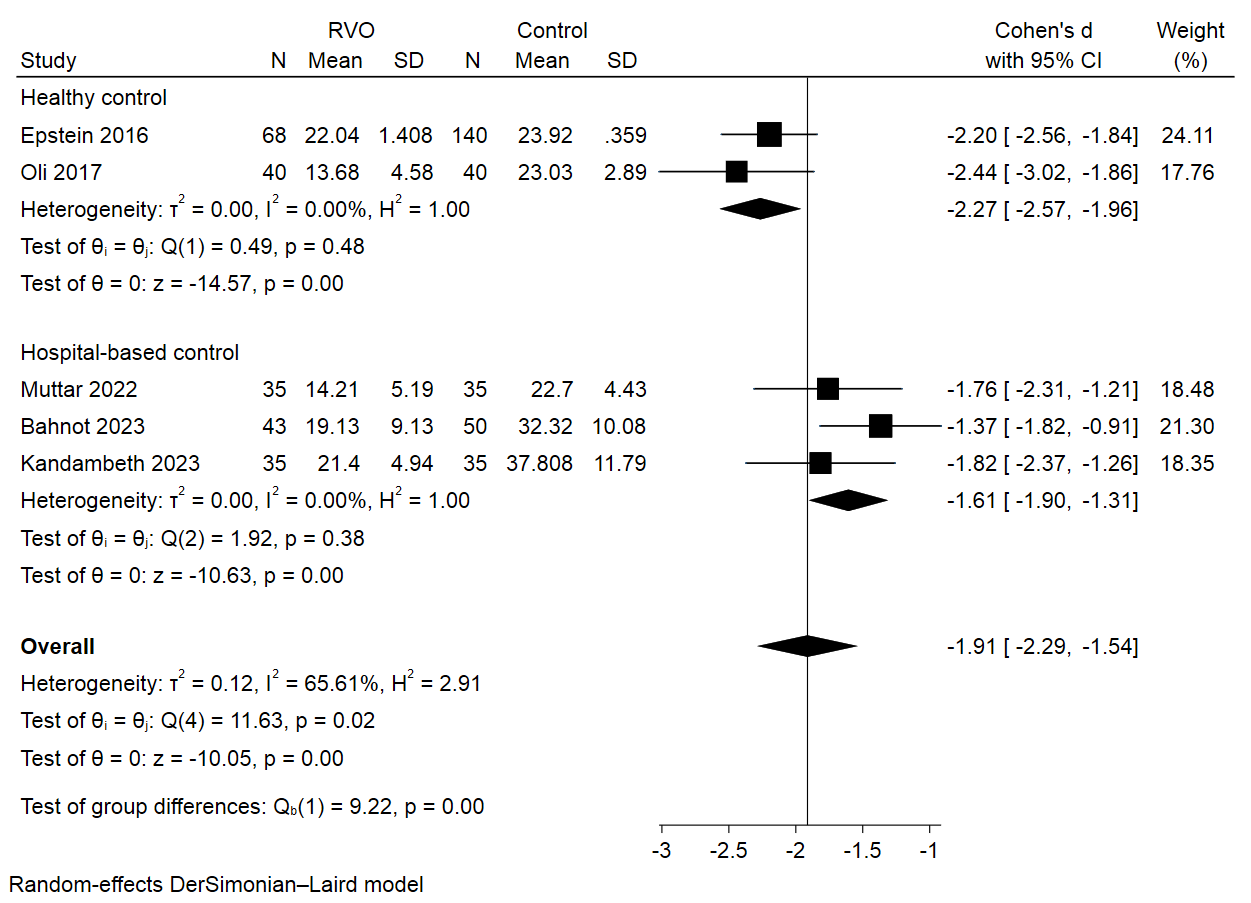


**Supplemental figure 4**. Forest plot of standard mean difference of vitamin D between RVO and controls according to the matching methodology based on seasonality


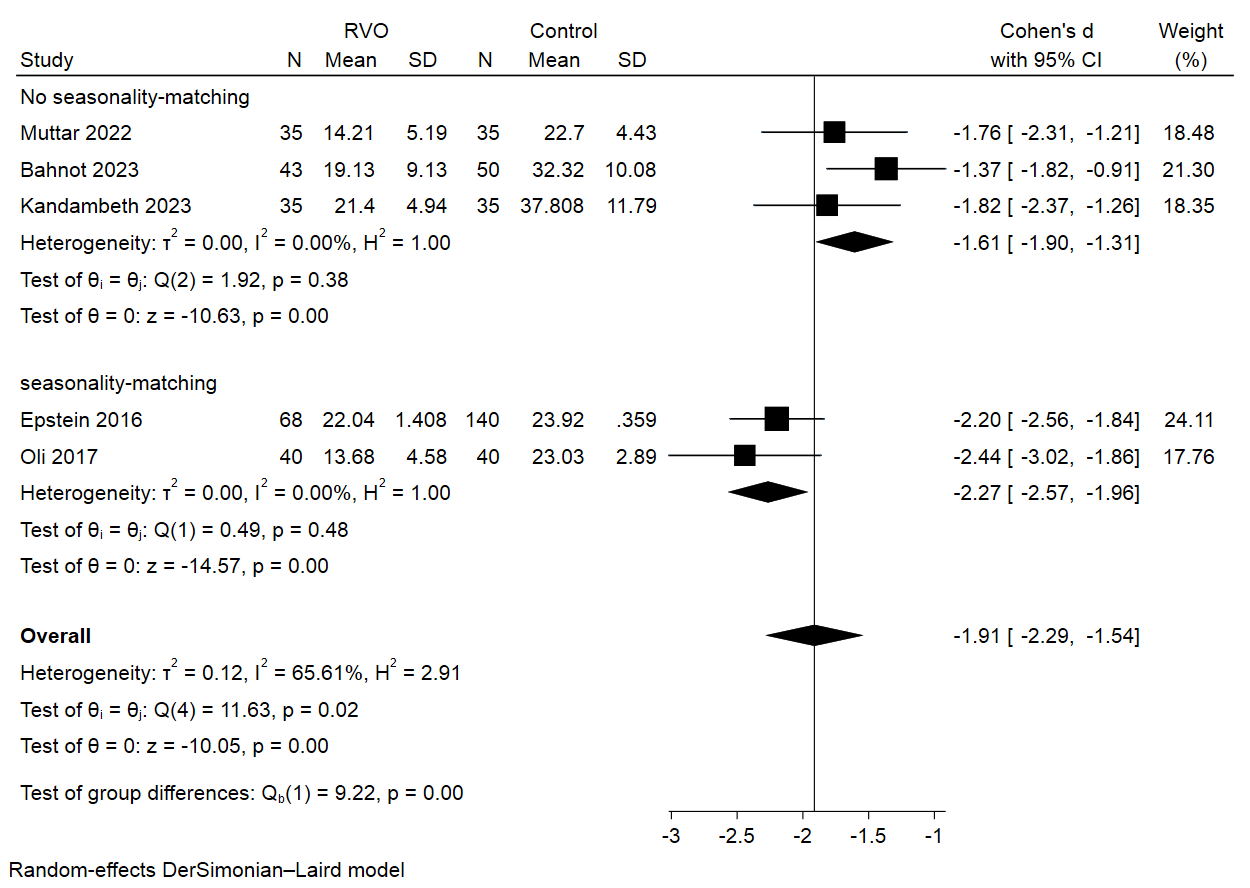


**Supplemental figure 5**. Forest plot of standard mean difference of vitamin D between RVO and controls according to the vitamin D measurement method


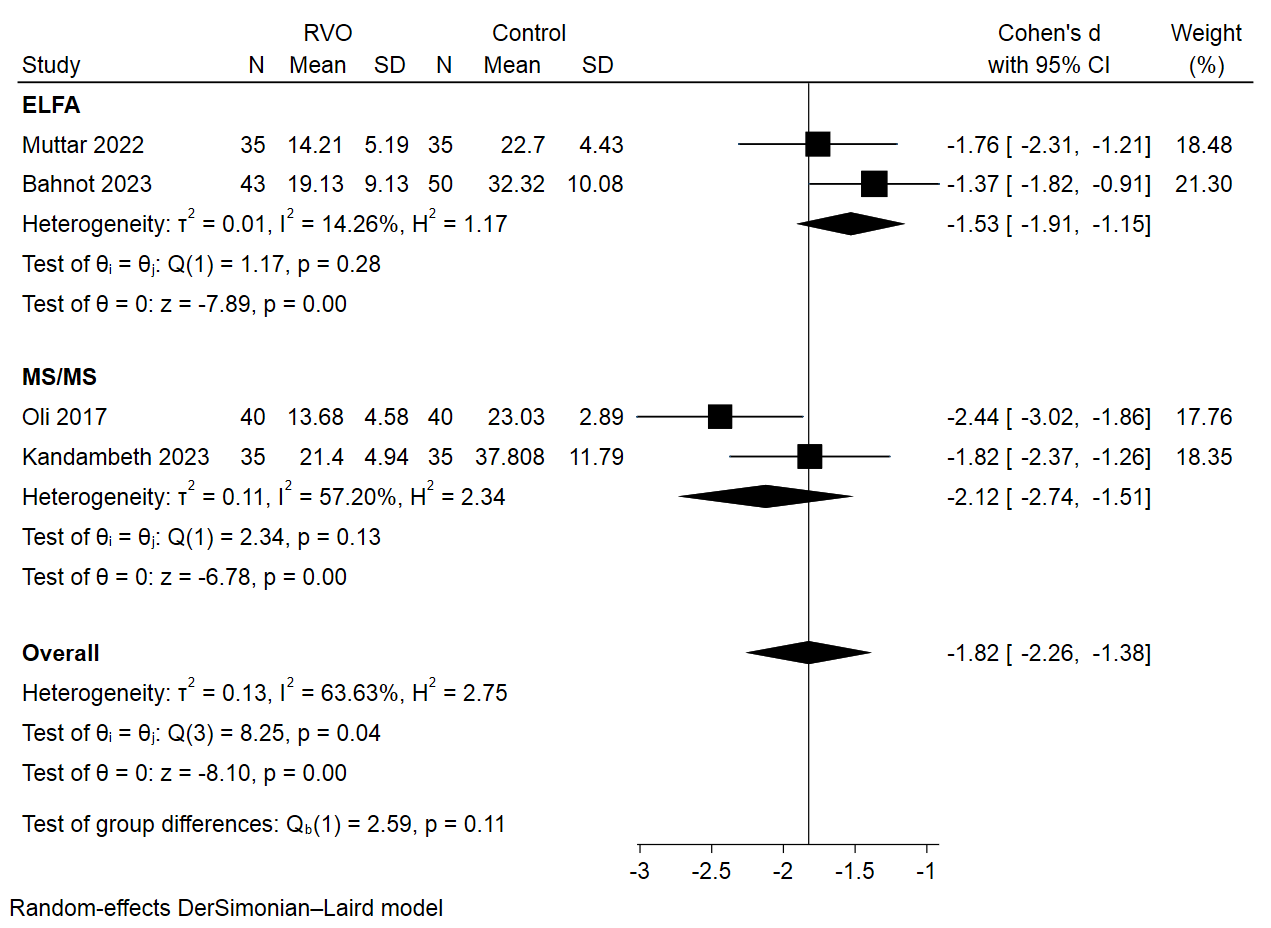


**Supplemental figure 6**. Forest plot of standard mean difference of vitamin D between RVO and controls according to the matching methodology based on sex


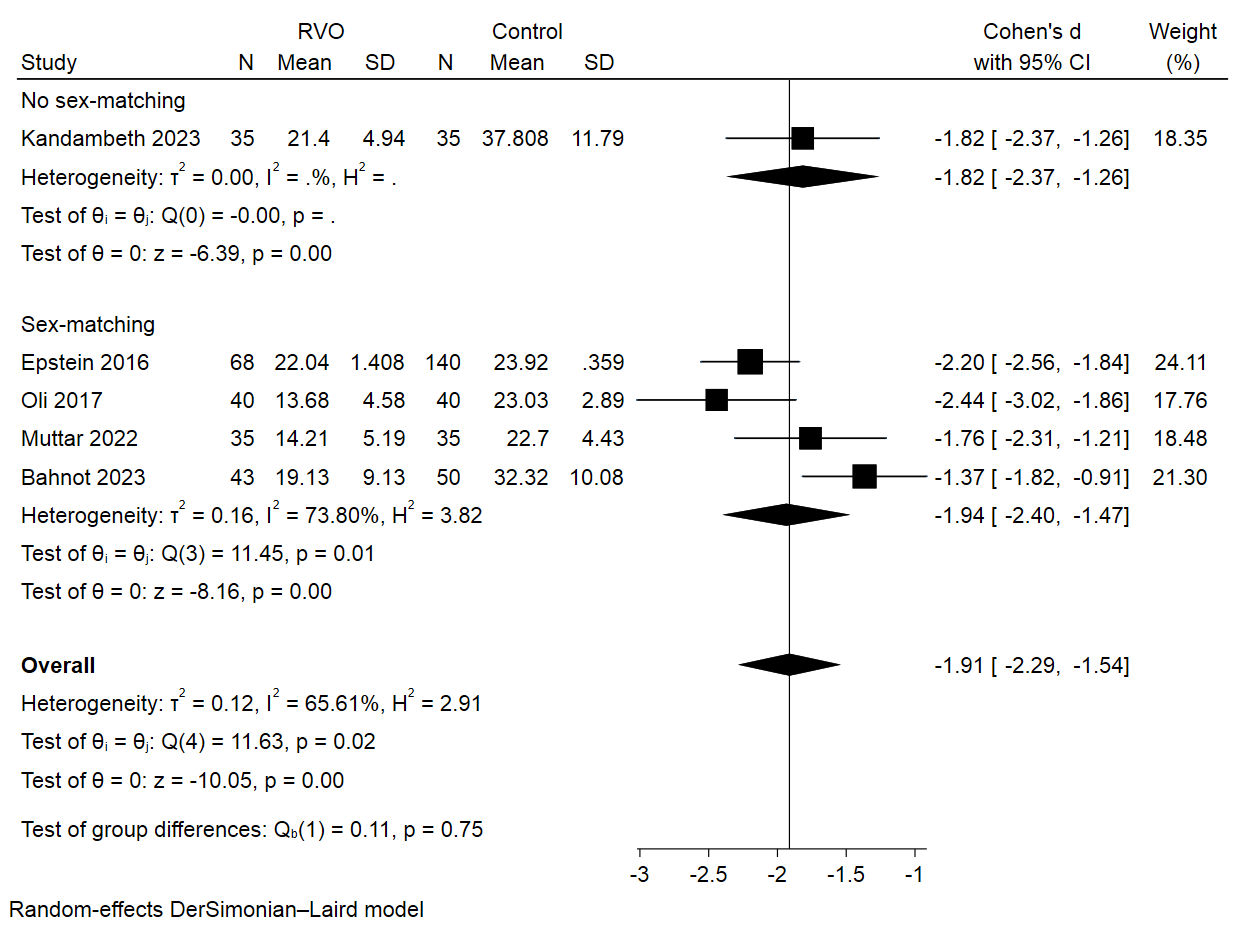


**Supplemental figure 7**. Forest plot of standard mean difference of vitamin D between RVO and controls according to the matching methodology based on diet


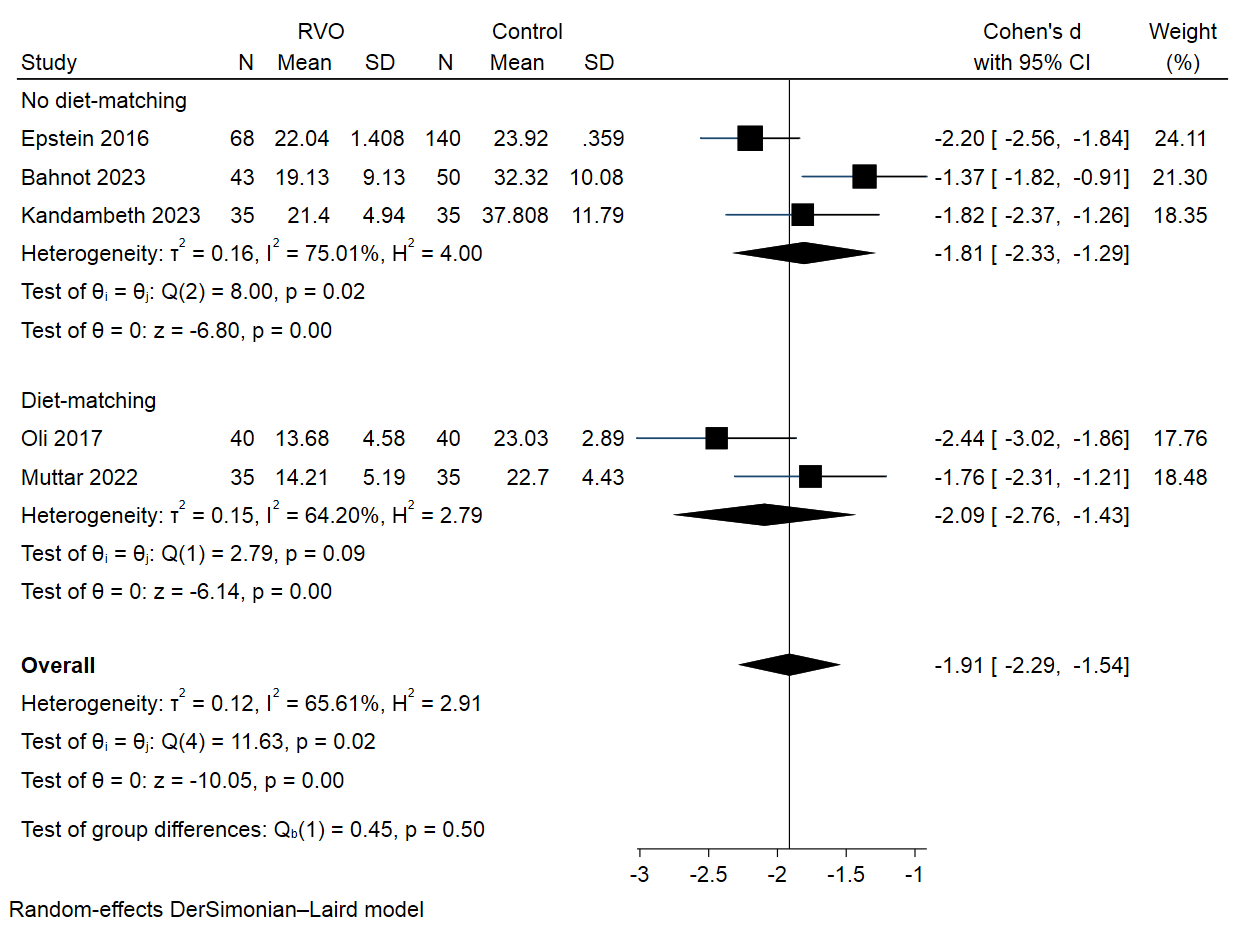


**Supplemental figure 8**. Forest plot of standard mean difference of vitamin D between RVO and controls according to the matching methodology based on country


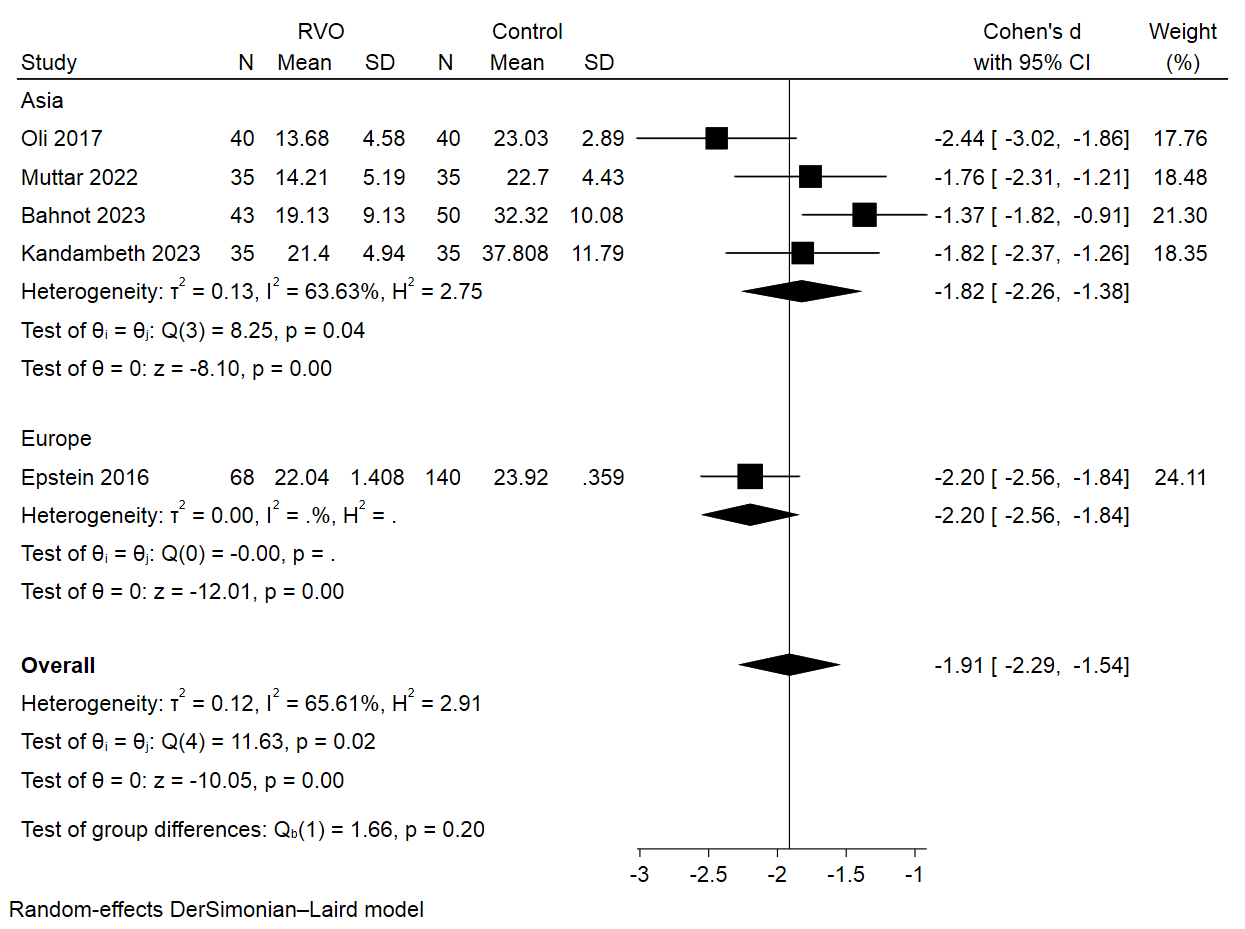


**Supplemental figure 9**. Funnel plot of studies. This funnel plot displays slight asymmetry, yet Egger’s and Begg’s tests reveal no significant publication bias (Egger’s P = 0.94, Begg’s P = 0.46).


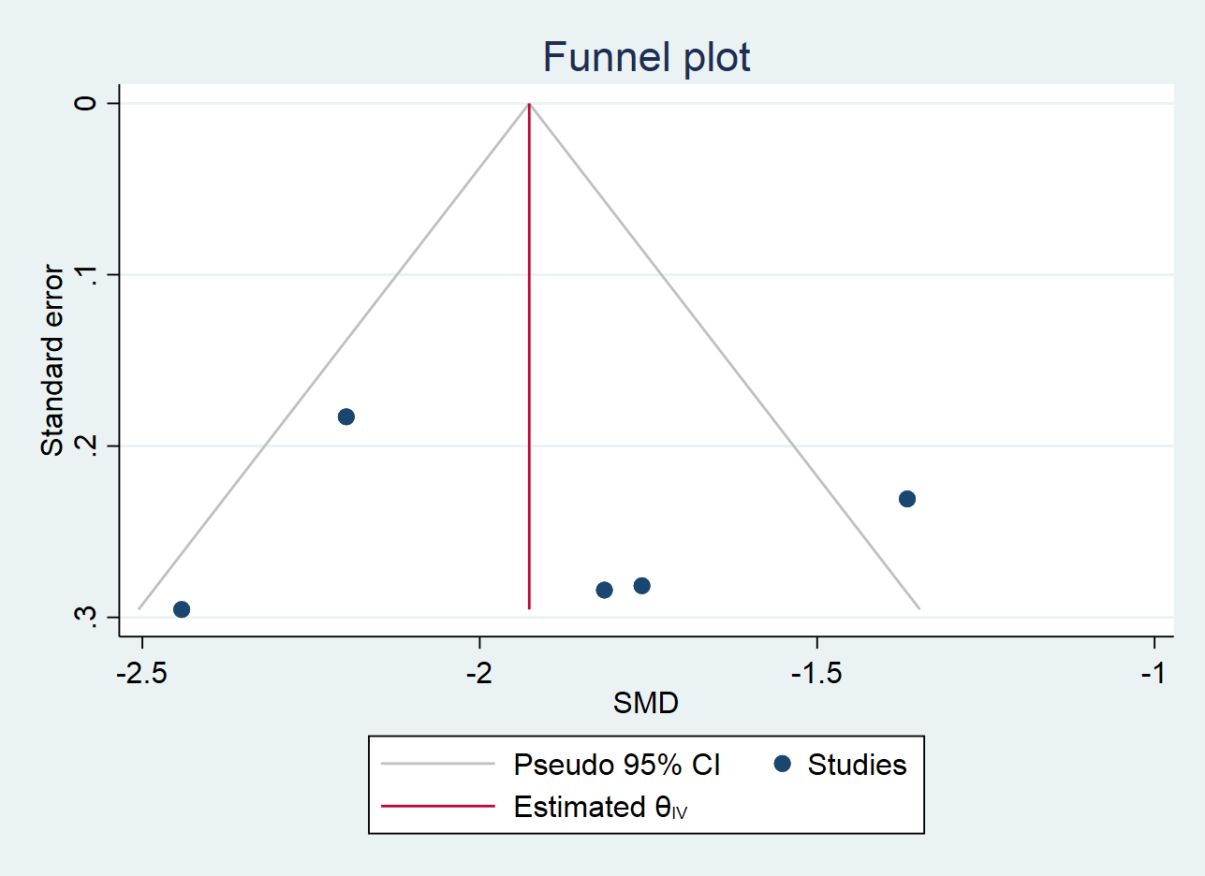


**Supplemental figure 10**. Forest plot of sensitivity analysis for prevalence of vitamin D deficiency. Study by Epstein et al, significantly influenced the pooled results.
